# A Study of the Laser Removal Process of Al-Si Coating from 22MnB5 Steel

**DOI:** 10.3390/ma16103709

**Published:** 2023-05-13

**Authors:** Tao Zhang, Jihao Xu, Shuxia Lin, Wangwang Yu, Yong Chen

**Affiliations:** 1 School of Mechanical Engineering, Nanjing Vocational University of Industry Technology, Nanjing 210023, China; 2 Associated Engineering Research Center of Mechanics and Mechatronic Equipment, Shandong University, Weihai 264209, China

**Keywords:** hot forming steel, 22MnB5 steel, Al-Si coating, laser removal, coating removal width

## Abstract

22MnB5 hot forming steel is widely used in the automotive industry due to the increasing demand for lightweight vehicles. As surface oxidation and decarburization occur during hot stamping processes, an Al-Si coating is often precoated on surfaces. The coating tends to melt into the melt pool during the laser welding of the matrix and reduce the strength of the welded joint; therefore, it should be removed. The decoating process by sub-nanosecond and picosecond lasers and process parameter optimization were conducted in this paper. The corresponding analysis of the different decoating processes, the mechanical properties and the elemental distribution was carried out after laser welding and heat treatment. It was found that the Al element has an influence on the strength and elongation of the welded joint. The high-power picosecond laser has a better removal effect than the lower power sub-nanosecond laser. The best mechanical properties of the welded joint were obtained under the process conditions of 1064 nm center wavelength, 15 kW power, 100 kHz frequency, and 0.1 m/s speed. In addition, the content of the coating metal elements (mainly Al) melted into the welded joint is reduced with increasing coating removal width, which significantly improves the mechanical properties of the welded joints. Al in the coating rarely melts into the welding pool when the coating removal width is not less than 0.4 mm, and its mechanical properties can meet the automotive stamping requirements for the welded plate.

## 1. Introduction

Lightweight automobiles play important roles in terms of energy, environment, safety and other aspects [1,2,3,4]. High-strength steel sheets are commonly used in the manufacturing of car bodies to ensure safety and reduce weight. Hot-formed 22MnB5 steel has excellent mechanical properties and is widely used in the production of crucial parts such as A/B pillars and bumpers [5,6,7]. Initially, the material exhibits a ferritic–pearlitic microstructure with a tensile strength of about 600 MPa. After the hot stamping process, the component finally has a martensitic microstructure with a total strength of about 1500 MPa [5]. These parts are first laser welded together into laser-welded panels and then hot stamped, and surface oxidation and decarburization inevitably occur during the steel sheet hot stamping process. As a result, an aluminum-silicon (Al-Si) coating is often pre-plated on its surface in order to prevent this shortcoming.

The Al element in the Al-Si coating will melt into the melt pool during laser welding of the base material, which can greatly reduce the strength of the joint. Studies have shown that the Fe-Al intermetallic compounds can easily form because the Al-Si coating flows with the weld metal along the fusion line during laser welding. Lin et al. conducted an experiment to obtain full-penetration laser welds of Al-Si coated steel in conduction mode [8]. Yoon et al. examined the distribution of Al in the coated layer, which affects the microstructure in the fusion zone, and the influence of before-and-after hot stamping on phase transformations in the segregation zone [9]. Lin et al. compared Al-Si coated and uncoated 22MnB5 steel plates under laser welding and hot stamping conditions and found that ferrite segregation caused a significant reduction in the tensile properties of the coated joints after stamping [10]. Ferrite can form from Fe-Al intermetallic compounds during subsequent weld cooling and the material does not change even after hot forming, which causes a reduction in the mechanical properties of the weld [11,12].

To solve this problem, scholars have performed a lot of work on coating treatments [13,14,15]. Kang et al. [14] investigated the laser weldability of hot press-formed steels with and without Al-Si coating. They laser welded specimens using disc and fiber laser docking and overlapping joints and examined the effect of process parameters on the strength of the welds. Removal of the coating is the most direct method and it is currently adopted by most manufacturers. There are three main methods for removing Al-Si coatings on hot-formed steels, namely laser ablation, mechanical grinding and chemical reaction [16]. ArcelorMittal [17] proposed that the Al layer on the surface of the coating can be removed by a partial ablation technology, and the mechanical properties of the laser welded joint can meet the requirements of automobile manufacturing. Li et al. [18] have studied the ablation effect of nanosecond pulse laser parameters (i.e., pulse energy, pulse repetition rate and pulse number) on Al-Si coating, found that the mechanisms of nanosecond laser ablation include material evaporation and phase explosion, which are mainly determined by the laser frequency. Sun et al. [19] have studied pulsed wave (PW) fiber laser welding of 1.4 mm-thick Al-Si coated 22MnB5 steel sheets to investigate the effect of pulse modulation on reducing the segregation of coating element in fusion zone (FZ) and improving mechanical properties of welded joints. Xu et al. [20] have studied the Al content for the formation of δ-ferrite in the FZ after welding, and analyzed the distribution of Al in the FZ after hot stamping. There is little literature on the effect of the removal state of hot stamping steel coating on the structure and properties of the weld. Therefore, it is particularly important to explore the influence of the coating removal state on the welded plate.

In order to study the effect of different laser removal processes on the removal of the coating and then understand the influence of the removal process on the mechanical properties of the welded joint, sub-nanosecond and picosecond lasers were used to remove the Al-Si coating. The optimal removal width was determined by analyzing the mechanical properties and element distribution of the welded joint.

## 2. Experimental Procedure

**Figure 1:**
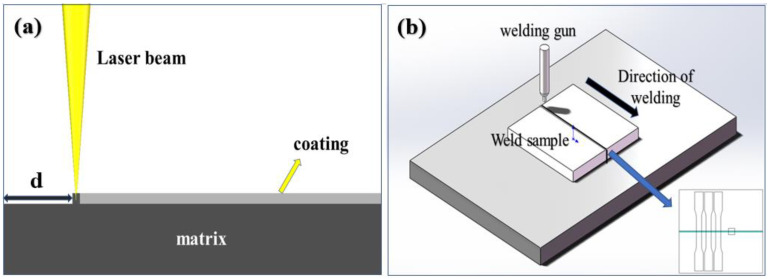
Experimental principles: (**a**) laser removal, (**b**) laser welding.

The material used in this study is commercial aluminum-silicon coating 22MnB5 steel with a thickness of 1.2 mm, specimen coatings of 15–24 µm, mainly pure aluminum (partially enriched in silicon); its chemical composition is shown in Table 1. The laser welding (TruDisk 4001, Suzhou, Jiangsu, China) is performed with a maximum power of 4 kW, a fiber core diameter of 400 μm, a focusing distance of 150 mm, and a spot diameter of 480 μm. Laser welding process parameters were unified using laser power 4 kW, welding speed 11.75 m/min, off-focus amount 0 mm, and kept warm at a temperature of 950 °C for 5 min with water cooling for 5–10 s after welding. The laser welding and laser removal process is shown in Figure 1, d is the width of the decoating in the figure. This experiment studies the removal process and removal effect under two different types of lasers (sub-nanosecond laser (Shanghai, China) and picosecond laser, TruMicro 5000, China); the corresponding parameters are shown in Table 2.

An optical microscope (Leica DMILM, Wetzlar, Germany) was used to observe the macroscopic morphology of the welded seam area and the surface of the plate after the laser decoating. WDW100 electronic universal testing machine was used for tensile testing of the welded joints along the direction perpendicular to the weld itself, with the tensile specimen spacing of 70 mm. A specimen with a gauge size of 50 mm × 12.6 mm is shown in Figure 1b. A scanning electron microscope (ZEISS Supra 55, Wetzlar, Germany) equipped with an EDS (Oxford Inca, Abingdon, UK) was used to perform a line scan of the upper and middle parts of the welded joint, and the elemental energy spectrum of the scanned area was analyzed.

## 3. Results and Discussion

### 3.1. The Laser Decoating Process

**Figure 2:**
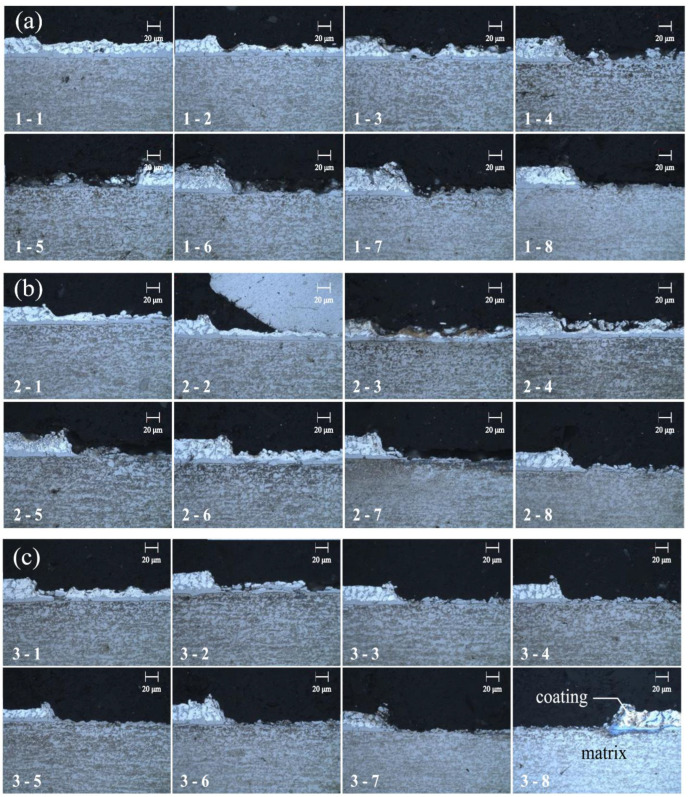
Metallographical diagram of laser removal test: (**a**) A1 group, (**b**) A2 group, (**c**) A3 group.

This experiment aimed to investigate the impact of laser power, laser frequency and scanning times on the mechanical properties of the welded plate after laser stitching. A sub-nanosecond laser was used for laser decoating experiments with a fixed power of 15 W, a scanning speed of 1000 mm/s, a spot size of 50 μm, a laser frequency of 50 kHz or 100 kHz, and a parallel linear continuous scanning mode. The 100 kHz high-frequency (HF) and the 50 kHz low-frequency (LF) scan numbers (labeled group A) were controlled to investigate the effect on the depth of decoating. The specific experimental parameters are shown in Table 3 and the micrographs can be observed in Figure 2.

As can be seen from Figure 2, the depth of decoating was gradually increased with an increasing number of HF scans in the A1 test group when the number of LF scans was 1. The number of HF scans was fixed at 3 for the A2 group of trials and the depth of decoating was progressively increased as the number of LF scans increased each time. However, the increases in decoating depth are limited, which indicated that the LF scan has a limited effect on the decoating depth. It mainly cleans the surface of the coating metal and removes residual surface coating metal. In the A3 group, it gradually increased with the number of LF scans with a fixed 4 times HF cleaning. It was found that the coating depth is also gradually increased and the material surface tended to be smooth. However, compared to the A2 group, the number of HF scans increased by one and the depth of the decoating layer increased significantly, which indicated that HF scanning is more effective for layer removal. The best de-coating results were obtained with the parameters of 4 HF scans and 8 LF scans in the groups A3-8.

**Figure 3:**
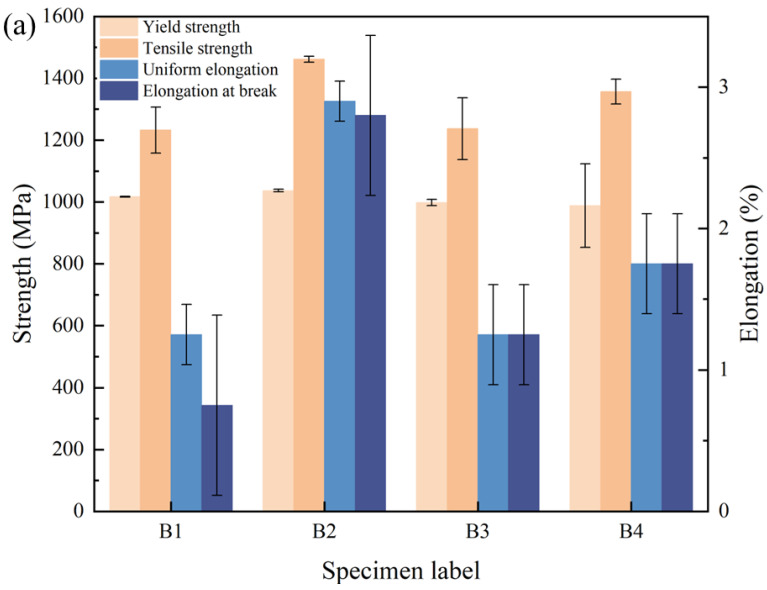

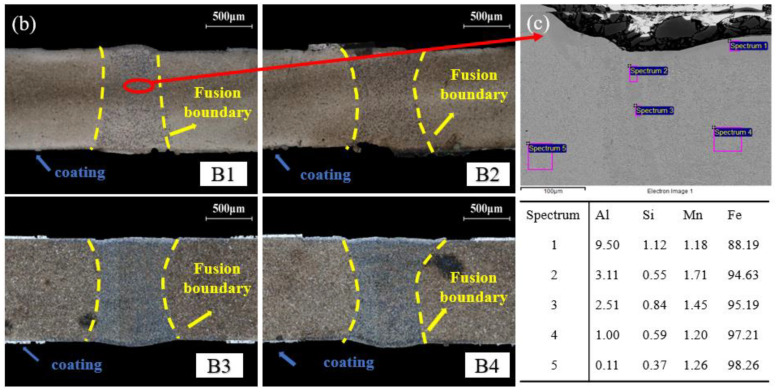
Analysis results of B group welded joints: (**a**) mechanical properties, (**b**) metallographic diagram, (**c**) EDS Analysis.

The A3-8 group parameters were chosen to conduct a decoating test with a 1 mm de-coating width. This was performed to investigate the mechanical properties of the welded joint. After welding and heat treatment, tensile tests were carried out to study the mechanical properties and the morphology of the weld seam. Group B specimens were welded to four welded plates, and three tensile specimens were taken from each plate to measure the average of their mechanical properties (Figure 3a). As can be seen from the graph, the tensile strength and the elongation fluctuates greatly under the same laser removal parameters, with strengths just above and below 1200 MPa and tensile rates below 2%, with the lowest dropping by nearly 70% compared to the base material. The possible reason is that the coating was not removed completely and residual coating metal still entered the weld. Further investigation is needed to better understand the factors that affect the effectiveness of the removal process and improve the quality of the welded joint.

The microstructure evolution is shown in Figure 3b. It is obvious that the color of the welded joint and the matrix are different, indicating a difference in composition. To further investigate the weld and matrix composition, the B1 specimen was selected for EDS analysis and the result is shown in Figure 3c. It was found that a large amount of Al element existed in the weld surface. The possible reasons are as follows: firstly, part of the melting material surface may be caused by the low peak power and large single pulse width of the nanosecond laser, which impaired the mechanical properties of the joint when removing the surface coating for a metal compound between Al and iron. Secondly, the metal dust may be generated and gather on the workpiece surface during the decoating process, which will cause a partial loss of laser power and a change in the laser focus position.

**Figure 4:**
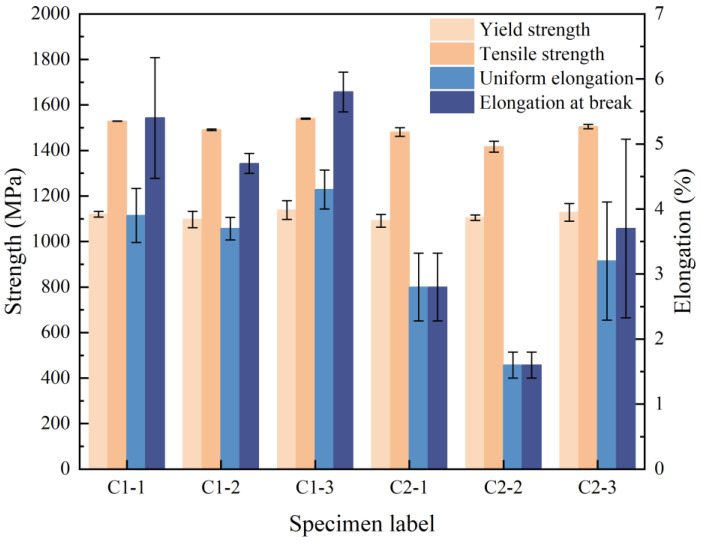
Mechanical properties test results.

As mentioned above, the research findings suggest that a laser with a higher peak power effectively vaporizes the plated metal instantly, thus minimizing the occurrence of a molten state and reducing the intrusion of the plated metal into the substrate. For this reason, a picosecond laser was chosen for the coating removal test (Group C) due to its narrow pulse width, higher single-pulse energy, and shorter heating time. These factors work synergistically to inhibit the tendency of the metal material to melt and to avoid the residue of compounds on the surface of the sheet due to the melting of the base material and plated metal. The experiments were carried out using both a primary removal (C1) process and a removal + cleaning (C2) process by the picosecond laser; the corresponding parameters are shown in Table 4. Three plates were welded with the same set of parameters and three tensile specimens were assessed in order to ensure the repeatability of the experiment; the results are shown in Figure 4.

**Figure 5:**
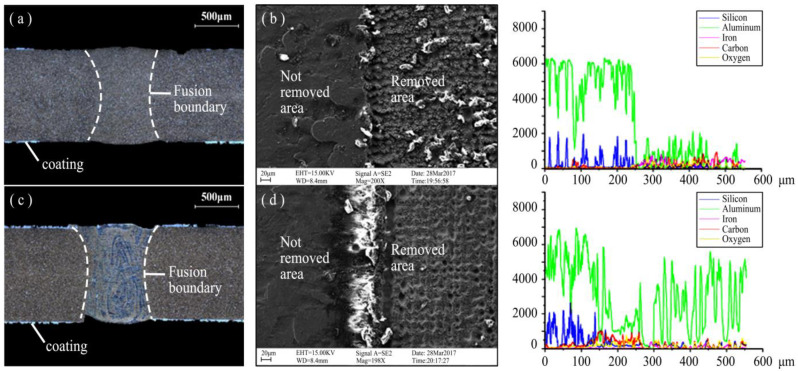
Microstructure analysis of welded joints: (**a**) C1 metallographic diagram, (**b**) C1 EDS, (**c**) C2 metallographic diagram, (**d**) C2 EDS.

As can be seen in Figure 4, C1 exhibited tensile strength and elongation meeting the mechanical properties of the product, with a tensile strength of over 1500 MPa and an elongation at break of over 4%. In the C2 group, the yield strength and tensile strength basically meet the requirements of the mechanical properties of the product, while the elongation index is still not satisfactory. It is shown that the C1 group obtained a better result than C2. However, there were still large fluctuations in the elongation measured on each plate. To further investigate the cause, metallographic tests were carried out on the welded joint sections and the results are shown in Figure 5.

It can be seen from Figure 5a that the microstructure of the matrix and welded joint is basically the same and the fusion boundary is no apparent, which indicates that the coating metal did not enter the matrix. Compared to C1, the welded joint and matrix of C2 in Figure 5c exhibited a difference composition. EDS analysis of the coating removal surface in Figure 5b,d showed that the coating removal effect is achieved by the C1 process for plates with a very low surface Al content. However, the surface achieved by the C2 process still has an unevenly distributed small amount of Al, which caused the formation of metal compounds in the welding process and the reaction of Fe. This is the reason that the mechanical properties of welded joints decreased. After the C1 removal process, the smaller amount of Al in the weld still caused the elongation to fluctuate, indicating that elongation is more sensitive to the amount of Al relative to strength.

In conclusion, the above findings show that the higher power picosecond lasers can remove the coating more effectively than nanosecond lasers. Using a one-time removal method, the C1 process combination using a high power of 15 W and a slow speed of 0.1 m/s had a better effect than the C2 process combination of removal + cleaning.

### 3.2. Effect of Laser Decoating Width on the Welded Joints

**Figure 6:**
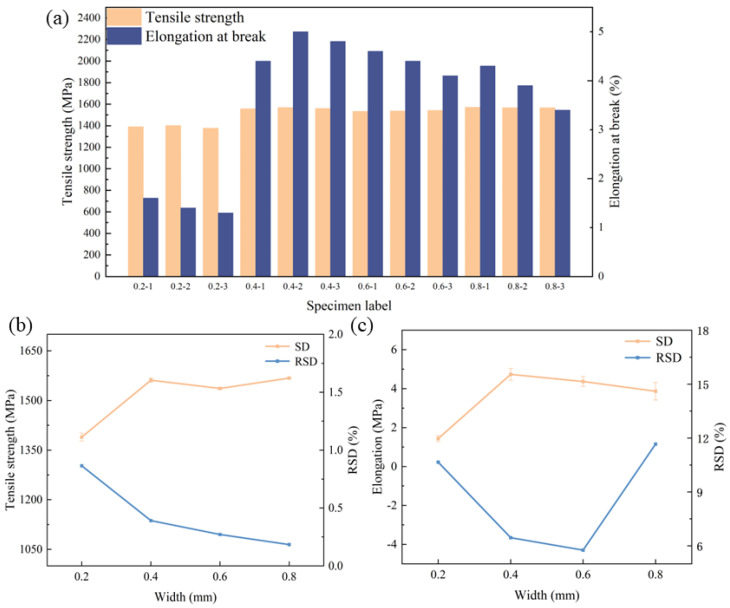
Tensile properties of welded joints: (**a**) tensile properties, (**b**) RSD of tensile strength, (**c**) RSD of elongation.

In order to study the influence of the decoating width on the mechanical properties of welded joints, the properties of welded joints were analyzed in terms of tensile strength, metallography, hardness and weld composition distribution. The minimum value for the decoating width of the weld was obtained to meet the requirements of the mechanical properties of the joint and improve production efficiency. The decoating width (d) was set 0.2 mm, 0.4 mm, 0.6 mm and 0.8 mm, and the laser parameters was selected according to C1 in Table 4. The mechanical properties of the welded specimens with different removals of plating width were tested and the results are shown in Figure 6.

From Figure 6a, it is clear that the mechanical properties of welded joints with a coating width of 0.2 mm are poor, with tensile strength less than 1400 MPa and fracture elongation less than 2%. The reason may be that the removal width is too small and the plated metal enters the weld during the welding process, which leads to a reduction in the mechanical properties of the welded joint. However, when the removal width is increased to no less than 0.4 mm, the tensile strength improves significantly and is above 1500 MPa, while the fracture elongation also increases to above 4%. This proves that the coating metal on the substrate has not been able to enter into the weld. It was shown that the average values of tensile strength and elongation significantly increased with the increase in removal width. Furthermore, the tensile strength gradually stabilizes as the width of the de-coating increases, indicating an optimal removal width. Nonetheless, the elongation to fracture still fluctuates considerably, suggesting that there may be other factors affecting the mechanical properties.

From Figure 6b,c, it can be seen that the relative standard deviation (RSD) values of the tensile strength of the joints with different removal widths are less than 1%. However, for the elongation to fracture, the RSD values increase significantly and the data are unstable, hinting the possibility of an unstable laser output triggered by the wide removal width employed. Another reason could be imperfect parallelism of the plate with the laser head during laser decoating, which leads to variations in the amount of defocus, rendering the removal of the coating different at different locations on the same plate. This results in local enrichment of the plated metal and the subsequent reduction in mechanical properties, compounding inconsistent mechanical properties throughout the plate. These findings further indicate that the plated metal is particularly sensitive to fracture elongation.

**Figure 7:**
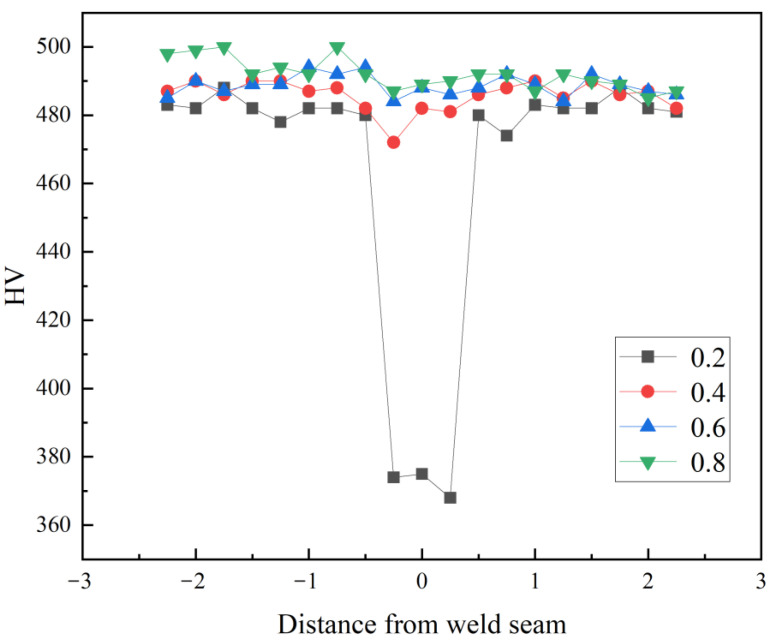
Hardness measurement results.

The metallographic and hardness analyses were carried out for further analysis on each group of specimens, and the hardness measurements of the welds and base material are shown in Figure 7.

As can be seen from Figure 7, the weld hardness values basically tend to the parent material and the weld area hardness value almost no decline except for the width of 0.2 mm. In order to further investigate the content and distribution of the coating metal, EDS analysis was carried out separately on welded joints of each decoating width.

**Figure 8:**
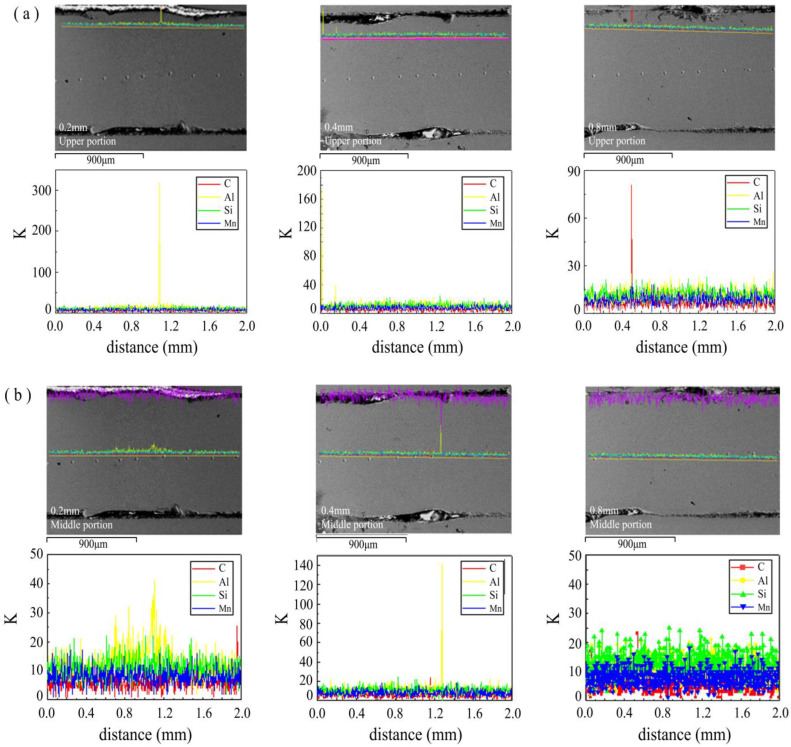
Elemental distribution of welded joints with different decoating widths: (**a**) upper part, (**b**) middle part.

**Figure 9:**
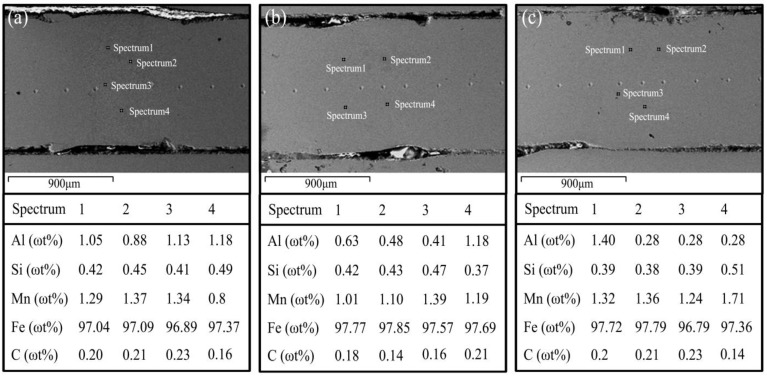
Elemental content analysis of welded joint with different decoating widths: (**a**) 0.2 mm, (**b**) 0.4 mm, (**c**) 0.8 mm.

The results of Figure 8 indicate that, aside from the 0.2 mm removal width, other removal widths of the welded joint exhibit almost no Al distribution. However, there is an enrichment of aluminum in some areas of the joint. This enrichment may potentially explain the variation of fracture elongation observed in welded joints. In order to specifically analyze the variation in the elemental aluminum content of the joints, the spot element content analysis (Figure 9) was carried out for each of the different de-plated widths of the welded joints.

As shown in Figure 9, the average content of the Al element for the 0.2 mm removal width is about 1~1.5 times higher than the other removal widths, which is the main reason for the decreasing tensile strength. When the content of Al element in the welded joint was kept at 0.4–0.6%, the joint tensile strength can reach more than 1500 MPa, but the elongation is unstable. The elongation is more stable with the 0.4 mm removal width, but the elongation has fluctuations at 0.8 mm. Large fluctuations in the elongation at break of welded joints of the same plate are due to uneven removal of the plating resulting in the enrichment of Al element in the welded joint, thus making the mechanical properties of the joint uneven and causing local performance degradation.

## 4. Conclusions

(1) High-power picosecond lasers are more effective for removing coating than low-power sub-nanosecond lasers. Under the removal parameters of 1064 nm center wavelength, 15 kW power, 100 kHz frequency and 0.1 m/s speed, the mechanical properties of the welded joint meet the application requirements.
(2) The content of coating metal elements (mainly Al) melted into the welded joint is reduced with the increasing coating removal width, which can significantly improve the mechanical properties of the welded joints.
(3) The Al element of the coating rarely melts into the melt pool when the coating removal width is not less than 0.4 mm, and its mechanical properties can meet automotive stamping requirements.

## Figures and Tables

**Table 1:** Chemical composition of the 22MnB5 steel (wt.%).

C	Si	Mn	Al	Ti	Cr	B	Fe
0.22	0.27	1.12	0.035	0.004	0.15	0.018	Bal.

**Table 2:** Comparison of laser parameters.

Parameter Lasers	Center Wavelength	Average Power	Repetition Frequency	Pulse Energy	Peak Power	Pulse Width
Sub-nanosecond	1064 nm	50 W	50 kHz	1 mJ	10 kW	100 ns
Picosecond	1064 nm	100 W	400 kHz	250 μJ	31.25 mW	8~10 ps

**Table 3:** Sub-nanosecond laser coating removal experimental parameters.

No.	HF	LF	No.	HF	LF	No.	HF	LF
A1-1	2	1	A2-1	3	1	A3-1	4	1
A1-2	3	1	A2-2	3	2	A3-2	4	2
A1-3	4	1	A2-3	3	3	A3-3	4	3
A1-4	5	1	A2-4	3	4	A3-4	4	4
A1-5	6	1	A2-5	3	5	A3-5	4	5
A1-6	7	1	A2-6	3	6	A3-6	4	6
A1-7	8	1	A2-7	3	7	A3-7	4	7
A1-8	9	1	A2-8	3	8	A3-8	4	8

**Table 4:** Picosecond laser test parameters.

No.	Average Power	Frequency	Speed	Times	Function
C1	15 W	100 kHz	0.1 m/s	1	removal
C2	12.5 W	100 kHz	0.3 m/s	2	removal
	10 W	100 kHz	1 m/s	1	cleaning

## Data Availability

Not applicable.
